# Bayesian phylogenetic analysis of Philippine languages supports a rapid migration of Malayo-Polynesian languages

**DOI:** 10.1038/s41598-024-65810-x

**Published:** 2024-06-28

**Authors:** Benedict King, Simon J. Greenhill, Lawrence A. Reid, Malcolm Ross, Mary Walworth, Russell D. Gray

**Affiliations:** 1 Department of Linguistic and Cultural Evolution, Max Planck Institute for Evolutionary Anthropology, 04103 Leipzig, Germany; 2 School of Biological Sciences, University of Auckland, Auckland, 1142 New Zealand; 3 National Museum of the Philippines, 1000 Ermita, Manila, Metro Manila Philippines; 4 Department of Linguistics, University of Hawaiʻi at Mānoa, Honolulu, HI 96822 USA; 5 School of Culture, History and Language, Australian National University, Canberra, 2601 Australia; 6 School of Psychology, University of Auckland, Auckland, 1142 New Zealand

**Keywords:** Language phylogenies, Language evolution, Linguistics, Austronesian, Migration, Philippines, Evolution of language, Phylogenetics

## Abstract

The Philippines are central to understanding the expansion of the Austronesian language family from its homeland in Taiwan. It remains unknown to what extent the distribution of Malayo-Polynesian languages has been shaped by back migrations and language leveling events following the initial Out-of-Taiwan expansion. Other aspects of language history, including the effect of language switching from non-Austronesian languages, also remain poorly understood. Here we apply Bayesian phylogenetic methods to a core-vocabulary dataset of Philippine languages. Our analysis strongly supports a sister group relationship between the Sangiric and Minahasan groups of northern Sulawesi on one hand, and the rest of the Philippine languages on the other, which is incompatible with a simple North-to-South dispersal from Taiwan. We find a pervasive geographical signal in our results, suggesting a dominant role for cultural diffusion in the evolution of Philippine languages. However, we do find some support for a later migration of Gorontalo-Mongondow languages to northern Sulawesi from the Philippines. Subsequent diffusion processes between languages in Sulawesi appear to have led to conflicting data and a highly unstable phylogenetic position for Gorontalo-Mongondow. In the Philippines, language switching to Austronesian in ‘Negrito’ groups appears to have occurred at different time-points throughout the Philippines, and based on our analysis, there is no discernible effect of language switching on the basic vocabulary.

## Introduction

Approximately 4500 years ago, Austronesian languages spread from their homeland in Taiwan to Island South-East Asia, and then expanded further into New Guinea, Oceania, parts of mainland South-East Asia and Madagascar^1,2^. These Austronesian languages outside of Taiwan all belong to the Malayo-Polynesian (MP) subgroup^3,4^. Based on archaeological data, it is widely accepted that the initial MP expansion was extremely rapid^5,6^. This rapid expansion would have left little time for linguistic innovations to arise and, therefore, few features with which to diagnose language subgroups. A rapid expansion is thus one possible explanation for the difficulty in finding relationships for the multiplicity of subgroups high in the MP tree^7,8^. Several scenarios have been proposed regarding the spread of Malayo-Polynesian languages out of Taiwan. There is disagreement over the extent to which languages have remained in place following this original migration or whether present-day language distributions result from a series of secondary migrations or population expansions^9^. It has also been proposed that the MP expansion actually consisted of many different migrations in different directions^10^. The archaeological data potentially supports the latter hypothesis, with approximately simultaneous age estimates for archaeological sites in the Philippines and Borneo. However, the archaeological record is too poorly sampled, and the date estimates too imprecise, to clearly distinguish between the alternative hypotheses^11^.

Austronesian speakers were not the first people in Island South-East Asia (ISEA). Hunter-gatherer populations are thought to have arrived in the Philippines at least 47,000 years ago^12^, with possible evidence of human occupation 67,000 years ago^13,14^. Relict populations of these original inhabitants remain in the Philippines, including the Aeta of Luzon, Batak of Palawan, Ati of Visaya and the Mamanwa of Mindanao. These groups are often collectively referred to as ‘Negritos’ or ‘Black Filipinos’^15^, although they are only very distantly related to each other. All ‘Negritos’ speak Austronesian languages to which they presumably shifted from distantly related and now-extinct indigenous languages. It is debated to what extent these ‘Negrito’ languages retain a substrate from their original languages. Reid^16^ suggested that unique lexical items in the Austronesian languages of ‘Negritos’ are potential retentions from their original, replaced languages. Languages of ‘Negritos’ are clearly Austronesian, but often appear only distantly related to languages spoken by their non- ‘Negrito’ neighbors^17,18^, suggesting that they were acquired early in the MP expansion^18^.

Whether or not there were populations other than ‘Negritos’ in ISEA prior to the MP expansion is more uncertain and, therefore, so are the relative roles of demic and cultural diffusion in the MP expansion. There is evidence that pre-Austronesian populations in Mainland South-East Asia switched to Austronesian from Austro-Asiatic languages^19,20^, although a small amount of demic diffusion was detectable in Cham populations^19^. In ISEA, Hill, et al.^21^ found that only a small proportion of mitochondrial DNA in ISEA could be linked directly with an Out-of-Taiwan dispersal. Austronesian-speaking populations in ISEA show a mixture of Austronesian and Austro-Asiatic genetic material^22^, although the relative timing of admixture events is often hard to determine. However, Admixture History Graphs based on the relative co-variance of ancestry components^23^ suggest that Papuan-Austroasiatic admixture occurred before the Austronesians arrived in Wallacea^24^. Further, genetic data from rice crops also challenges the idea that Austronesian expansion was a demographic dispersal associated with agriculture because rice varieties from the Philippines show more affinity with those from Mainland South-East Asia than those from Taiwan^25^. Larena, et al.^26^ suggest that the ancestors of today’s Austronesian-speaking ethnic groups in the Philippines arrived in several waves between 15,000 and 8000 years ago, again before the accepted ~ 4500 BP date for the Out-of-Taiwan dispersal.

One hypothesis to explain these discrepancies is that there were Austro-Asiatic speakers in ISEA prior to the MP expansion, and these populations switched to speaking Austronesian languages^27^. It has been argued that the combination of lexical conservatism and grammatical disparity in MP languages is symptomatic of this type of widespread language shift^28^. It has also been suggested that the Land Dayak MP languages of Borneo show evidence of an Austroasiatic substrate^29^, but this evidence is disputed^30^. Archaeological data is sparse, and much of the evidence for Austroasiatic peoples in Indonesia prior to Austronesian expansion centers around the archaeological site at Gua Sireh. Pottery from this site has carved, cord-wrapped or basket-wrapped paddle-impressed surfaces, more similar to mainland archaeological assemblages than the red-slipped pottery associated with Austronesians^31^. Although the Gua Sireh site has also been suggested to show evidence for early rice agriculture, a more detailed analysis of the pottery sherds revealed no evidence of domestic rice^32^.

The Philippines, lying immediately south of Taiwan and forming the probable first stepping stone in the expansion^33^, are central to debates regarding the dispersal of MP languages. There have long been arguments in favor of a discrete Philippine subgroup of MP, based primarily on lists of putative lexical innovations^34–36^. This hypothesis is associated with an agricultural-demographic expansion, whereby a single language, Proto-Philippines, emerged sometime after the initial colonization of the Philippines and replaced the remaining languages in the Philippines as its speakers sought new agricultural land, an event often known as a “language leveling episode”^35,37^. Such a demographic expansion would explain the relatively low language disparity within the Philippines^38^ and reconciles the proto-Philippines hypothesis with the expected rake-like language phylogeny that would be expected to emerge from a rapid Out-of-Taiwan expansion^7^.

However, there is an ongoing debate about the validity of the Philippine subgroup. The quality of most of the proposed lexical innovations has been challenged because they are not distributed across all Philippine microgroups^7,39,40^. Smith^7^, in reviewing the evidence for Proto-Philippines, concluded that only *bulbul (feather) and *dakə́l (big) might be true replacement innovations. Ross^39^ questioned why a proto-language with so many putative lexical innovations did not also show evidence of phonological or grammatical innovations. Ross^39^ tentatively suggested that the languages of the Batanic islands, an island chain between Taiwan and Luzon, might form the first branch of MP and descend from the first Austronesian people to leave Taiwan, but has since abandoned this idea^40^. Although a phonological merger of Proto-Austronesian *z and *d has been argued to define the Philippine languages^36,41^, it is attested in several Formosan subgroups^42^ and absent in the Central Luzon subgroup^43^, suggesting it is of little diagnostic value.

The relationships among the languages in the Philippines remain an open question. Blust^44^ identifies 15 Philippine micro-groups: Bashiic/Batanic, Cordilleran/Northern Luzon, Central Luzon, Inati, Kalamian, Bilic, South Mangyan, Palawanic, Central Philippines (including Tagalog, Bisayan, Bikol, Mansakan), Manobo, Danau, Subanon, Minahasan, Sangiric and Gorontalo-Mongondow. The latter three groups are languages of northern Sulawesi, which are included within the putative Philippine language subgroup. Seven Philippine micro-groups were hypothesized by Blust to form a single higher-level subgroup of Greater Central Philippines (GCP): Central Philippines, South Mangyan, Palawanic, Manobo, Danau, Subanon and Gorontalo-Mongondow. The Greater Central Philippine subgroup is suggested to be the result of a second language levelling episode, similar to the first Philippines-wide event^37^. GCP is supported by lexical innovations and the merger of Proto-Philippines *g and *R^44^. The inclusion of Gorontalo-Mongondow within the Greater Central Philippine subgroup is significant as this micro-group is located in northern Sulawesi, south of the Sangiric and Minahasan languages, suggesting the GCP expansion bypassed these micro-groups.

Phylogenetic methods are increasingly used to uncover and evaluate language relationships. Gray, et al.^45^ used Bayesian methods on a dataset of Austronesian languages. Regarding the Philippines, they found no support for Greater Central Philippines, with the Gorontalo-Mongondow languages instead forming the sister group to the other Philippine languages However, the sampling of languages in this analysis was non-representative, and the cognate-coding for these Philippines languages was incomplete. Since the publication of Gray, et al.^45^, the Austronesian Basic Vocabulary Database^46^ on which it is based has seen a significant increase in the number of languages included and an overhaul of the cognate coding in Formosan and Philippine languages.

Here we apply Bayesian phylogenetics to Philippine languages data from a revised and updated Austronesian Basic Vocabulary Database. This data includes, for the first time, representatives of the Minahasan, Sangiric, Danao, Inati, Northern Mangyan and Manide-Alabat micro-groups. We aim, in particular, to establish the relationships of northern Sulawesi languages to other Philippine languages, determine the effect of language shift by ‘Negritos’ on lexical evolution and test the hypothesis of language leveling events within the Philippines. Our results are discussed with respect to hypotheses regarding the Out-of-Taiwan dispersal of MP languages.

## Results

### Summary of phylogenetic relationships

**Figure 1:**
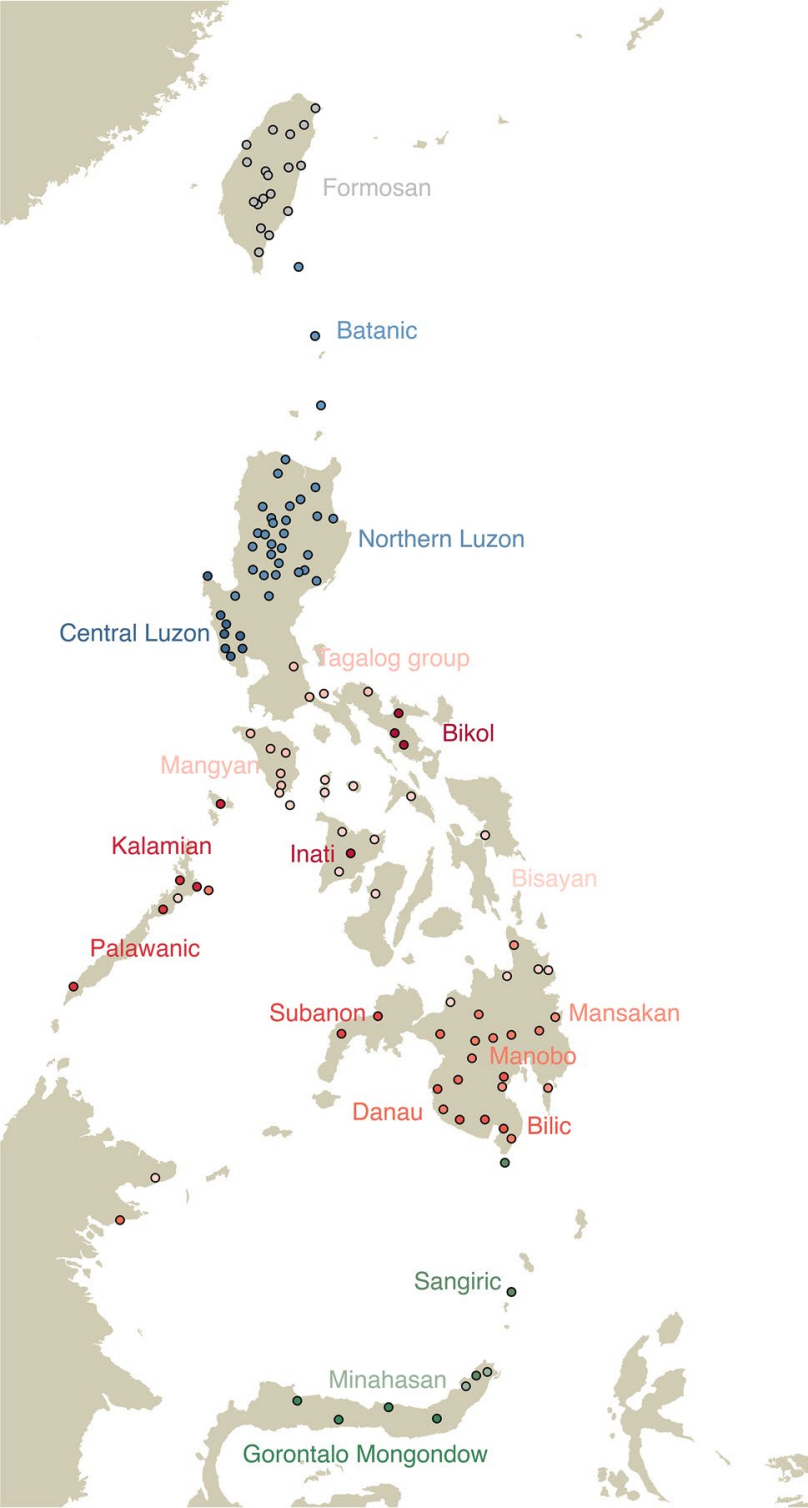
Locations of languages used in the phylogenetic analysis.

**Figure 2:**
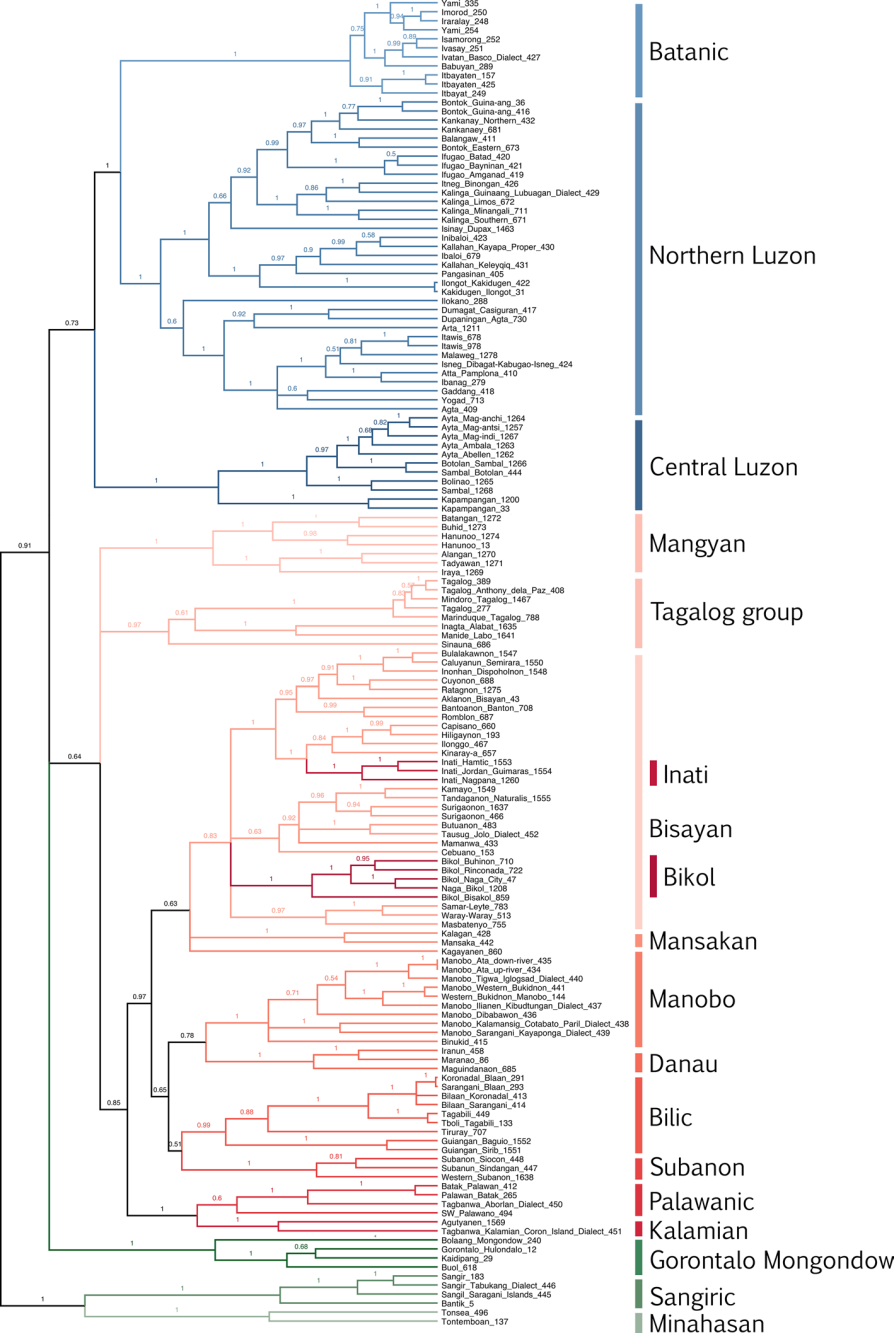
50% Majority-rule consensus tree of the phylogenetic analysis of Philippine languages. Formosan outgroup not shown on figure.

We performed a phylogenetic analysis in BEAST2^47^ of a basic vocabulary dataset of 7565 cognate sets in 185 meanings for 202 Formosan and Philippine doculects. The locations of the sampled doculects are shown in Fig. 1. The summary tree (50% majority-rule consensus tree) of the main analysis is shown in Fig. 2.

We find strong support for a group composed of the Sangiric and Minahasan languages (posterior probability (pp) = 1.0), which is in turn, the sister group to the other Philippine languages (pp = 0.91). We reconstructed lexical innovations on the branch defining the Philippine languages to the exclusion of Sangiric-Minahasan. This revealed eight probable innovations (with posterior probabilities > 0.5; Table 1). Two of these (branch_2, man_male_2) can be discounted as they are found outside the Philippines in other Malayo-Polynesian languages not included in our sample. The other six however appear to be legitimate and geographically widespread innovations.

**Table 1:** Cognate sets estimated to be innovations in the languages of the Philippines proper and Gorontalo-Mongondow, which together initially diverged from the Sangiric and Minahasan languages of northern Sulawesi. The number in the cognate set name indicates the cognate code from the Austronesian Basic Vocabulary Database.

Cognate set	Posterior probability of innovation
Branch 2	0.78
Earth/soil 25 *lutáq (ACD)	0.62
Sea 25	0.62
To dream 30	0.61
Man/male 2	0.61
Fruit 9	0.59
When 10	0.53
Who 14	0.5

We find strong support for a number of higher-level subgroups within Philippines. Northern Luzon and Batanic languages are grouped together (pp = 1.0), as are the Northern and Southern Mangyan subgroups (pp = 1.0), Tagalog with a handful of ‘Negrito’ languages (Manide-Alabat and Sinauna) (pp = 0.97) and Palawanic with Kalamian (pp = 1.0). There is also evidence for a large Central/Southern Philippine group (pp = 0.97) consisting of the Inati, Bisayan, Bikol, Mansakan, Manobo, Danau, Bilic, and Subanon micro-groups. Kagayanen falls separately to the other Manobo languages in the consensus tree, and only groups with the others with a posterior probability of 0.19. The ‘Negrito’ Mamanwa language does not group with the other Mansakan languages (pp = 0.0). The Bisayan group is not supported, and is found to be paraphyletic with respect to Inati, Mamanwa, and Bikol.

**Figure 3:**
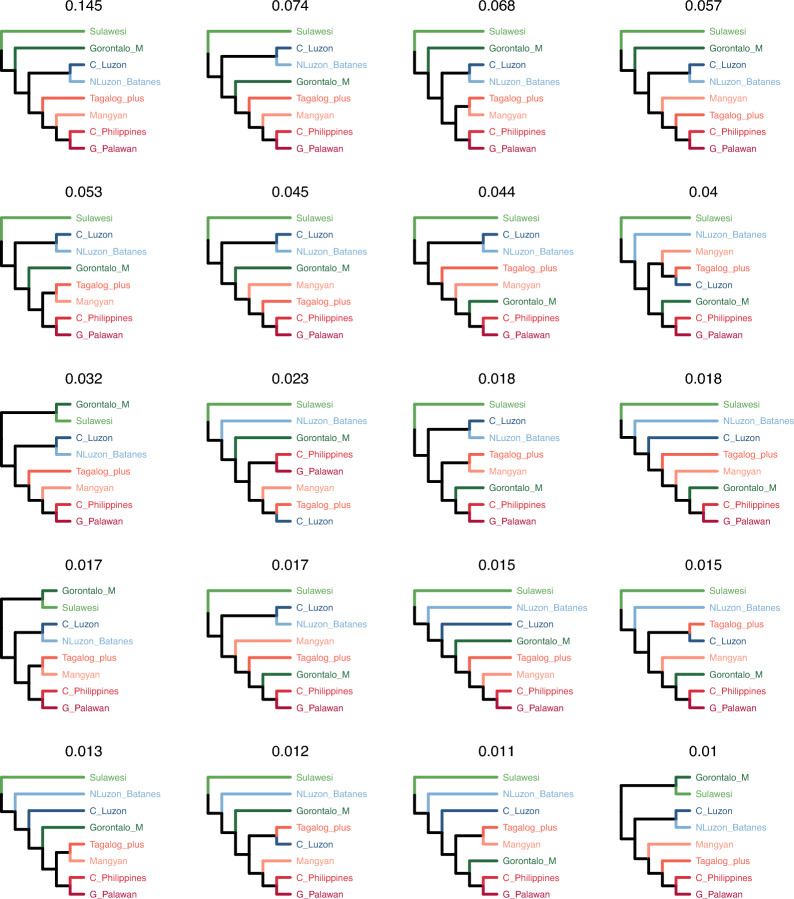
Summary of relationships between the major strongly supported Philippine subgroups from the posterior sample of trees, in descending order of support. The number above each tree indicates posterior support. The 20 tree topologies shown together represent a 73% credible set.

The relationships between six strongly supported higher level subgroups, and the Central Luzon and Gorontalo-Mongondow micro-groups, are highly uncertain. Alternative topologies for the interrelationships of these eight groups are shown in Fig. 3 in order of their posterior probability. Highlighting the uncertainty in these relationships, the single topology with the highest support has a posterior probability of just 0.15, and the top 20 topologies account for just 73% of the posterior sample. Much of this uncertainty is due to the positions of Gorontalo-Mongondow and Central Luzon. Gorontalo-Mongondow is found either as the second branch of the tree, as the sister group to the rest of the Philippine languages after the divergence of the Sangiric-Minahasan group, or nested somewhere with the Central Philippine, Palawanic, Mangyan or Tagalog languages. The Central Luzon languages are either found in a group with the Northern-Luzon and Batanic languages or with Tagalog.

### Greater Central Philippines and Gorontalo-Mongondow

We found no evidence for the Greater Central Philippine (GCP) subgroup as traditionally defined, which has a posterior probability of 0 in our analysis. However, if the GCP hypothesis is modified to also include the Bilic languages, we do find some support (pp = 0.43). Whether or not this modified GCP is supported is contingent upon the position of Gorontalo-Mongondow. It is found outside the rest of the Philippines as an early diverging branch with posterior probability 0.43, or groups somewhere with Central Philippine languages with posterior probability 0.56. Henceforth we will use “macro-GCP” to refer to the modified GCP hypothesis (i.e. including Bilic).

**Figure 4:**
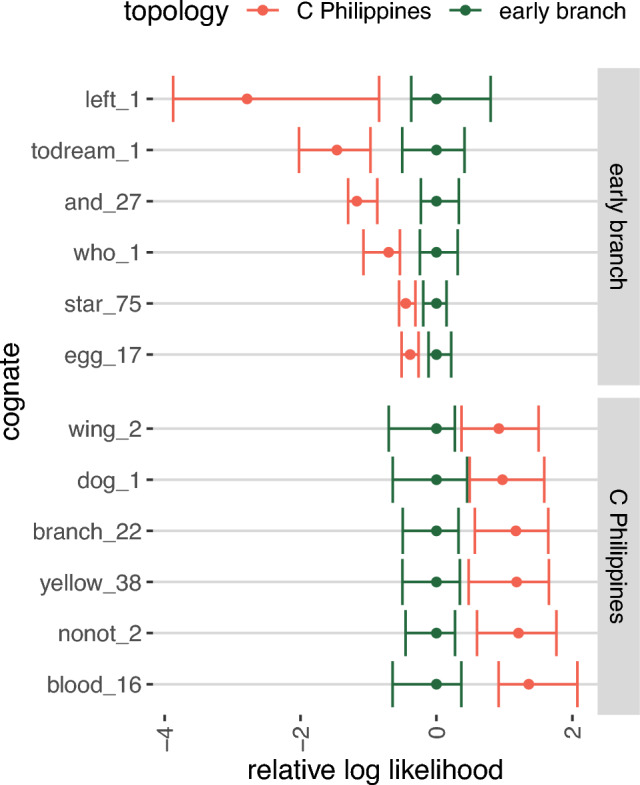
Cognate sets supporting either an early branching position for Gorontalo-Mongondow (top, left_1 to egg_17) or a Central Philippine (bottom, wing_2 to blood_16) position. 50% highest posterior density intervals of the likelihood of cognates in analyses with one of the two topologies constrained. Likelihoods are relative to the median in the early branching analysis. Only cognate sets with non-overlapping HPD intervals between the two analyses are shown.

We performed a topology test to reveal which lexical cognates provide evidence for the two conflicting positions for Gorontalo-Mongondow (Fig. 4), i.e. that Gorontalo-Mongondow forms an early branch of Philippine languages (diverging from other Philippine languages shortly after the divergence of Sangiric and Minahasan) or nests with Central Philippine languages. When we consider cognates with non-overlapping 50% Highest Posterior Density (HPD) intervals are considered, there are 6 cognates supporting each of the two topologies.

Cognate sets supporting an early branching position for Gorontalo-Mongondow are either widespread proto-MP forms, or forms shared exclusively with Sangiric and Minahasan languages. Left_1 (PMP *ka-wiʀi), todream_1 (PMP *hipi) and who_1 (PMP *i-sai) are found in Malayo-Polynesian languages not included in our analysis, and in all three cases are also not completely absent from the non-Sulawesi languages in our sample. We therefore do not consider these to be evidence for a core-Philippine subgroup. And_27 (e.g. Bantik bo), star_75 (e.g. Tonsea toti') and egg_17 (e.g. Bantik natúʔ) are exclusively shared between members of the Gorontalo-Mongondow, Minahasan and Sangiric languages within our dataset.

Two of the cognate sets supporting a Central Philippine position for the Gorontalo-Mongondow languages appear to be genuine innovations: blood_16 (proto-GCP *dugúq^44^) and yellow_38 (Proto-GCP *darág) are shared exclusively by Gorontalo-Mongondow and Central Philippine languages. Three other cognates supporting a Central Philippine position for Gorontalo-Mongondow are discounted because they are PMP forms found broadly in MP languages outside our sample: wing_2 (PMP *panid), dog_1 (PMP *asu) and no/not_2 (PMP (Zorc) *diaq). Branch_22 (e.g. Proto-Minahasan *paŋa) was possibly lost in macro-GCP.

### Language contact

**Figure 5:**
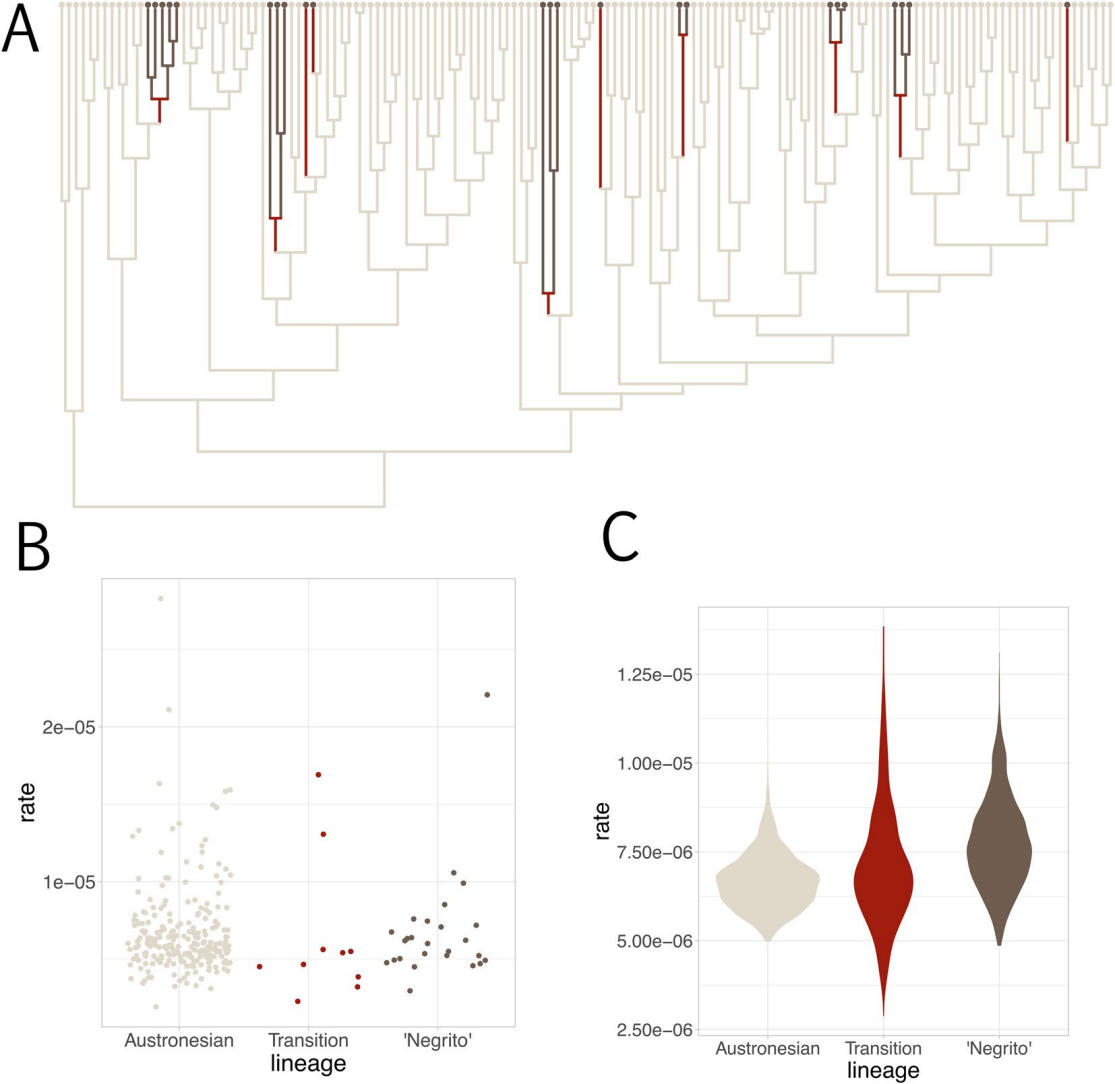
Language switching to Austronesian leaves no noticeable impression on the core vocabulary lexicon of the languages of ‘Negrito’ groups. (**A**) Phylogenetic tree (Maximum Clade Credibility tree) with branches on which language switching is inferred to have occurred highlighted in red. (**B**) Branch rates taken from the MCC tree (dot plot with random jitter). (**C**) Violin plot of the posterior distribution of the weighted mean rate of lexical evolution estimated on branches within Austronesian populations, within ‘Negrito’ populations and at the transition.

We identified 10 independent transitions of Austronesian languages to ‘Negrito’ people by performing an ancestral state reconstruction (Fig. 5A, Table 2). These transitions are dated to various times within the last 2000 years: the oldest transition (to Sinauna, Inagata Alabat and Manide Labo) is dated to a minimum of 1929 BP, and the youngest (Atta Pamplona) to a maximum of 437 BP (Table 2).

**Table 2:** Austronesian language switching to ‘Negrito’ populations. Identified by maximum parsimony ancestral state reconstruction on the MCC tree (Fig. 5A). Minimum and maximum ages (BP) represent the nodes bookending the branches on the MCC tree on which the transitions are inferred to have occurred.

Minimum	Maximum	Languages
1420	1652	Arta, Dumagat_Casiguran, Dupaningan_Agta
610	778	Ayta_Abellen, Ayta_Ambala, Ayta_Mag-indi, Ayta_Mag-anchi, Ayta_Mag-antsi
174	1005	Batak_Palawan, Palawan_Batak
585	1014	Inati_Nagpana, Inati_Hamtic, Inati_Jordan_Guimaras
225	713	Manobo_Tigwa_Iglogsad_Dialect, Manobo_Ata_down-river, Manobo_Ata_up-river
1929	2080	Sinauna, Inagta_Alabat, Manide_Labo
0	1141	Agta
0	437	Atta_Pamplona
0	907	Mamanwa
0	1220	Iraya

The Philippine languages spoken by ‘Negritos’ show no discernable impact of contact on the core vocabulary. For each tree in the posterior sample, we recorded the rates of lexical evolution on the branch in the phylogeny in which a transition from a non-Austronesian to an Austronesian language in a ‘Negrito’ population is inferred. These branches did not show elevated rates of lexical evolution when compared to the rest of the tree (Fig. 5).

## Discussion

We found no evidence of a North-to-South migration signal in the tree topology. In agreement with Ross^40^, the hypothesis that the languages of the Batanes islands represent a first-order branch of MP^39^ should be abandoned. Instead, our analysis shows a general pattern of South-to-North dispersal. This pattern rests principally on the deep branching position of the languages of northern Sulawesi and the nesting of Batanic within Luzon, both of which are strongly supported.

Our results show a dominant geographical signal. In our analysis, several groups considered to be only distantly related to other Philippine languages are strongly supported as sister groups to their geographic neighbors. The Kalamian micro-group, previously not considered a close relative of any other micro-group^48^, is grouped with Palawanic with strong support (pp = 1.0). The Northern Mangyan languages have been considered as relatives of the Central Luzon group^49,50^, but here we find strong support for a relationship with the Southern Mangyan languages, also from the island of Mindoro, in agreement with a lexicostatistical study^51^. Manide-Alabat, considered a deep branch of Philippines not closely related to any other^17^, is grouped with neighboring Tagalog along with Sinauna. Conversely Kagayanen, a geographically isolated outlier of the Manobo micro-group, does not group with other Manobo languages.

The dominant geographical signal in our results may partly be explained by an initial rapid Out-of-Taiwan dispersal producing a widely dispersed, undifferentiated Proto-Language. This scenario would be expected to initially produce a rake-like tree topology^7^, with later borrowing due to contact the only remaining signal in the data. This explains the recovery of the unusual groupings listed above.

In addition to the effects of borrowings, our results are also consistent with the hypothesis that the languages of the Philippines form a linkage^40,52^. This linkage effect is shown by the unstable relationships in the deeper branches of the tree, best demonstrated by the changing position of Central Luzon, which is generally grouped with Northern Luzon, but also sometimes with Tagalog (Fig. 3).

**Figure 6:**
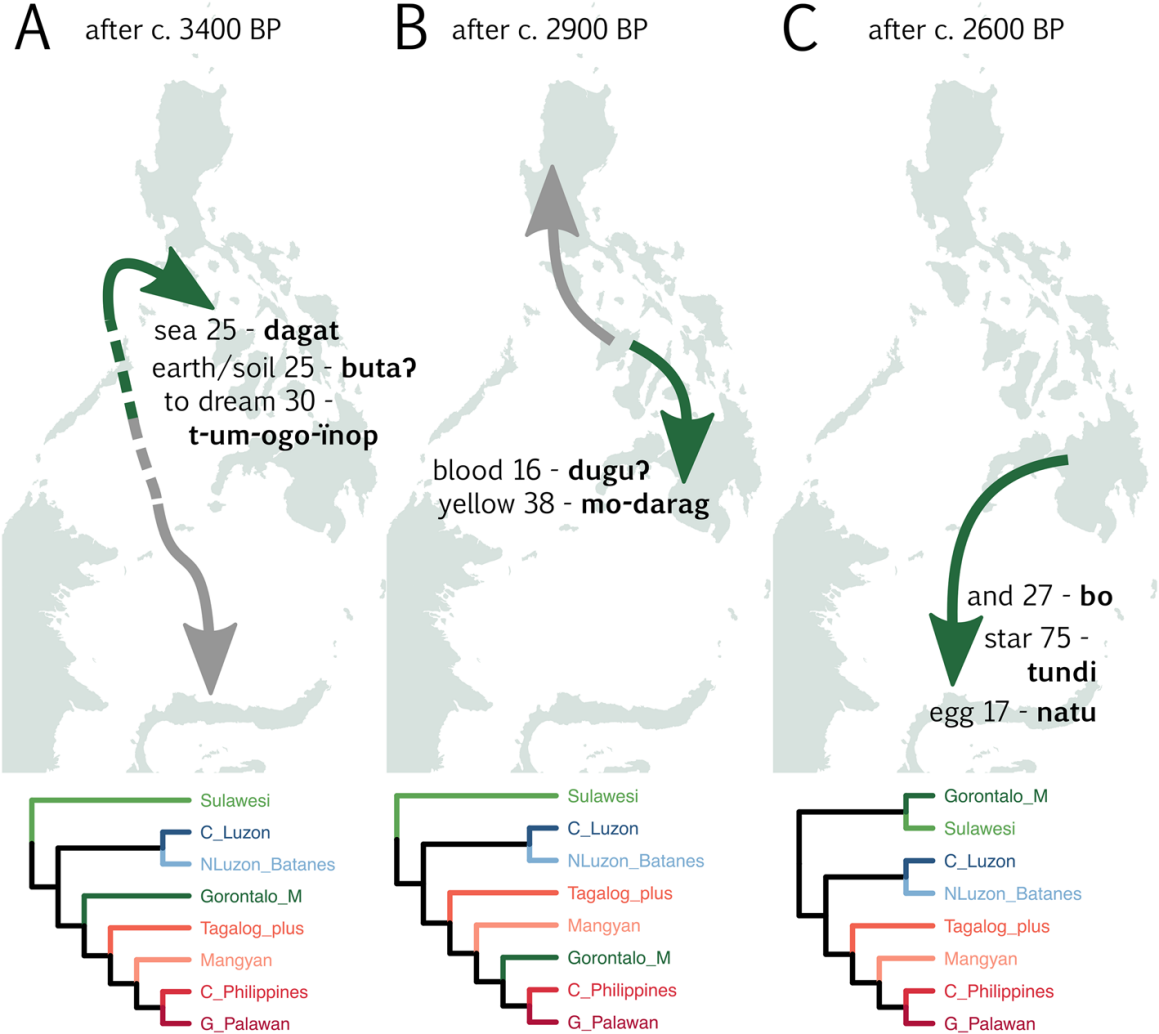
Scenario for the accumulation of the Gorontalo-Mongondow lexicon in three phases. Lexicon exemplified by the forms from Bolaang Mongondow. The bottom panels show examples of phylogenetic trees (one example for each panel, selected from Fig. 3) that each of the three levels of lexical data would support. (**A**) First divergence of Philippine languages; languages in the Philippines proper acquire cognate sets not found in Sangiric/Minahasan. (**B**) Philippine languages split into a Northern and a South-Central group (“Greater Central Philippines”). (**C**) Gorontalo-Mongondow migrates to northern Sulawesi, and shared cognate sets with Sangiric/Minahasan spread by diffusion. Dates based on median branching time of (**A**) Sangiric/Minahasan from other languages in trees where this is the first split (pp = 0.91), (**B**) Splitting of macro-GCP from other languages in trees where GCP is recovered (pp = 0.43) and C) splitting of Gorontalo-Mongondow and its closest relative, in trees where macro-GCP is recovered.

Gorontalo-Mongondow languages contain different layers of cognate sets, reflecting a possible migration event followed by diffusion between neighboring languages. The evidence from cognate sets (Fig. 4) is consistent with the following scenario (Fig. 6). First, during the initial spread of MP languages in the Philippines, the Sangiric and Minahasan micro-groups split from the rest of the Philippine languages. From this initial period, Gorontalo-Mongondow languages retain a number of cognate sets that are innovations shared with all the other Philippine languages except Sangiric and Minahasan (Fig. 6A). Later, the languages in the Philippines proper underwent a broad North vs South/Central split, during which time Greater Central Philippines cognate sets were acquired (Fig. 6B). In the final stage, People speaking Proto-Gorontalo-Mongondow migrated to northern Sulawesi^44^, following which cognate sets unique to the languages of northern Sulawesi spread by diffusion (Fig. 6C). The result is a conflict between different parts of the lexical data, some showing the historical signal, and others the geographic/diffusion signal. The latter pulls Gorontalo-Mongondow into an earlier branching part of the tree than it might otherwise be (Fig. 6, bottom panels), and leads to an unstable phylogenetic position (Fig. 3).

A similar migration-diffusion scenario is also a likely explanation for our inferred position of Kagayanen. Kagayanen is an outlier Manobo language, the only one not found on Mindanao. Although considered part of the Manobo subgroup, it also shows evidence of diffusion from Bisayan languages^53^. This conflicting signal likely explains its anomalously deep branching position in the consensus tree (Fig. 2), although some support still exists for uniting it with Manobo.

Conflicting data produce alternative phylogenetic positions based on historical and diffusion signals; in the case of Gorontalo-Mongondow, we interpret these to be with Central Philippines or with Sangiric/Minahasan, respectively. However, the conflicting data can also lead to “compromise” phylogenetic positions. In our posterior sample of trees, Gorontalo-Mongondow can branch off anywhere along the phylogenetic path between Sangiric/Minahasan and Central Philippines, and the consensus tree shows an unresolved, “compromise” position (Fig. 2). We predict that languages that have undergone a significant migration event will often appear in unusually deep branching positions in phylogenies due to conflicting signals from related languages on one hand and neighboring languages on the other.

The proposed language leveling event at the origin of Philippine languages is potentially supported by the long branch between the root of the tree and the divergence of Philippine languages: the branch length has a median length of 1219 years (HPD 471–2093), approximately a quarter of the tree depth, and the divergence of Philippine languages is dated at 3379 BP (HPD 2570–4208). This potentially provides some support for the Proto-Philippines hypothesis, but analysis of a wider sample of Malayo-Polynesian than that used here is required to directly test Proto-Philippines using Bayesian phylogenetics. Although we find some support for a variant of the Greater Central Philippine subgroup of Philippine languages, there is no evidence for the long branch leading to this group that a language levelling event would predict. We therefore find no evidence of a second language-levelling episode.

Our analysis reveals no conclusive signal of contact from language switching in ‘Negrito’ populations. It has been hypothesized that these languages retain words from a pre-Austronesian substrate^16^, including many in core vocabulary. In a phylogenetic analysis, retained lexemes would appear as innovations occurring on the branch on which language switching is inferred to have occurred. A substantial number of imported lexemes from a pre-Austronesian language would result in a rapid rate of lexical change being estimated on that branch. Although we find no evidence for such an increase in rates, our analysis does not rule out such retentions occurring at low frequency, outside core vocabulary, or in grammatical features. We also found no evidence that all ‘Negrito’ populations switched to MP languages at a relatively early stage, as previously hypothesized^18^. Instead, language switching appears to have occurred at various times in different languages (Table 2). The lack of evidence for early switching to Austronesian by ‘Negrito’ speakers suggests that there may have been minimal contact between Austronesian and ‘Negrito’ populations during the initial Out-of-Taiwan migration.

A lack of evidence for contact in ‘Negrito’ languages raises the probability that switching to Austronesian languages involved wholesale adoption of the lexicon with little retention from previous languages. This has potential implications for the spread of MP languages more generally, given that genetic data hints at a possible pre-Austronesian presence of East Asian peoples in ISEA. Whether MP languages result from a mixture of language-switching and demographic expansion or a purely demographic expansion, our results suggest that these two hypotheses may be difficult to distinguish based on lexical data, since uncontroversial examples of language switching in ‘Negritos’ leave no obvious pattern.

Our study uses several features available in the BEAST2 framework for linking the results of the analysis directly to the data that ultimately support it. We applied topology tests to examine the strength of evidence for and against alternative positions for Gorontalo-Mongondow, and also probabilistically reconstructed lexical innovations occurring on branches of interest. These kinds of analyses add an extra dimension to Bayesian analysis of linguistic data, allowing assessments about the strength of the evidence supporting a particular hypothesis beyond the posterior probability values returned by standard analyses.

Overall, our results conclusively reject a simplistic North-to-South dispersal of Austronesian languages in the Philippines. Instead, we propose an initial rapid expansion from the south, followed by high levels of diffusion across language chains, including repeated language shifts from ‘Negrito’ to Austronesian. Our investigation of the data also reveals substantial effects of contact on the distribution of lexical cognates. In contrast, there is little evidence for secondary demographic expansion and language levelling events beyond a possible event at the origin of Philippine languages and the migration of Gorontalo-Mongondow. This suggests a dominant role for cultural diffusion in the Philippines following Austronesian expansion. Our implementation of several methods to scrutinize the results of our Bayesian analysis serve as a template for Bayesian analysis of linguistic data in future studies.

## Materials and methods

We assembled a dataset of Philippine and Formosan languages from the Austronesian Basic Vocabulary Database^46^, discarding any doculects with less than 80 cognate sets. The sample consisted of 202 doculects: 147 from the Philippines and an outgroup of 55 Formosan doculects. The data comprised 7565 cognate sets in 185 meanings and is curated at https://github.com/SimonGreenhill/abvd_philippines. A table showing the languages and their sources is included (SI Appendix, Table S1). The cognate coding for Formosan and Philippine languages has been extensively revised since the analysis of Gray, et al.^45^ by L Reid (Philippines) and M Ross (Formosan).

The data were analyzed in BEAST2.7.3^47^. We applied the covarion model^54,55^, which allows cognates to switch between hidden states with slow and fast rates of change. We set the frequencies of the hidden states to (0.5, 0.5), the default in BEAST. The relative frequencies of states absent and present were set to their empirical values (0.992, 0.008). We applied an ascertainment bias correction because all-zero cognate sets are not observed^56^, and applied this correction separately to each meaning to account for differing patterns of missing data. Cognate sets in the Austronesian Basic Vocabulary Database can be divided into “primary cognate sets” and “sub-cognate sets”, with the latter encoding sound changes that modify an underlying primary cognate set. These subcognate sets remain incompletely or inconsistently coded and were therefore removed. The remaining primary cognate sets were partitioned into bins based on the number of cognate sets in each meaning, with bins of 1–10, 11–20 etc. cognate sets. We applied a yule model tree prior with a lognormal prior (− 7.5, 2.0) on the birth rate and a lognormal relaxed clock with a lognormal prior (− 10.0, 2.0) on the mean branch rate and a gamma distribution (0.5396, 0.3819) on the standard deviation. The prior on the root of the tree was a normal distribution on 5200 years BP, with a standard deviation of 100, based on the results of Gray, et al.^45^.

We ran the analysis for 100 million generation across 3 independent runs. Convergence was confirmed in Tracer^57^, with ESS > 200 for all parameters. The log files from the 3 runs were combined following the removal of 10% burn-in.

We performed two types of tests to determine cognate sets supporting various topological relationships. The first was a topology test^58^, to examine alternative phylogenetic positions for Gorontalo-Mongondow. We ran one analysis with a constraint enforcing Gorontalo-Mongondow to fall outside the languages of the Philippines proper, i.e. an early branching position close to Sangiric and Minahasan. We performed a second analysis with Gorontalo-Mongondow constrained to group with Central/Southern Philippine languages. We recorded the log likelihood of each cognate set throughout the MCMC in both the analyses. The Highest Posterior Density Interval of each cognate set was then calculated separately for each analysis. Cognate sets with non-overlapping 50% HPD intervals between the two analyses were then considered as significant, supporting one or the other phylogenetic position for Gorontalo-Mongondow.

In the second test, we estimated the lexical innovations occurring in the lineage leading to a group of interest (e.g. all Philippine languages excluding Sangiric and Minahasan). We performed ancestral state reconstruction of all cognate sets at the most recent common ancestor (MRCA) node of the group of interest, as well as at the immediately ancestral node (also known as the origin node). Cognate sets reconstructed as absent (covarion states 0 or 2) at the origin node and as present (covarion states 1 or 3) at the MRCA node can be designated as innovations, which were logged to an output file using a newly formulated beast2 java class (InnovationLogger) curated at https://github.com/king-ben/innovations. These reconstructions are performed at every sampled generation of the MCMC chain, thereby leading to a probabilistic estimation: the posterior probability of a particular innovation is the proportion of the sampled generations in the MCMC in which an innovation of a cognate is reconstructed.

### Supplementary Information

Supplementary Information.

## Data Availability

Data and analysis code are available at 10.6084/m9.figshare.22347916.
