# Supplementary material for: Bayesian phylogenetic analysis of Philippine languages supports a rapid migration of Malayo-Polynesian languages
Source: Sci Rep. 2024 Jun 28;14:14967. doi: 10.1038/s41598-024-65810-x (PMC11213883; doi:10.1038/s41598-024-65810-x)
Supplement: Supplementary file 1 — Supplementary Information. [file 41598_2024_65810_MOESM1_ESM.docx]

Supplementary Table S1. List of doculects from the Austronesian Basic Vocabulary Database that were used in the phylogenetic analysis.

| ABVD | ISO-639-3 | Glottocode | Name | TreeName | Source |
| --- | --- | --- | --- | --- | --- |
| 5 | bnq | bant1286 | Bantik | Bantik_5 | Theddon (2005) |
| 12 | gor | goro1259 | Gorontalo (Hulondalo) | Gorontalo_Hulondalo_12 | Machmoed (2005) |
| 13 | hnn | hanu1241 | Hanunóo | Hanunoo_13 | H. C. Conklin (1953) |
| 29 | kzp | kaid1239 | Kaidipang | Kaidipang_29 | Usup (2005) |
| 31 | ilk | ilon1239 | Kakiduge:n Ilongot | Kakidugen_Ilongot_31 | Reid (1971) |
| 33 | pam | pamp1243 | Kapampangan | Kapampangan_33 | Forman (1971);McFarland (1977) |
| 36 | lbk | guin1256 | Bontok, Guina-ang | Bontok_Guina-ang_36 | Reid (1971) |
| 43 | akl | akla1241 | Aklanon - Bisayan | Aklanon_Bisayan_43 | D. Zorc (2005) |
| 47 | bcl | cent2087 | Bikol (Naga City) | Bikol_Naga_City_47 | Daniega (2005) |
| 86 | mrw | mara1404 | Maranao | Maranao_86 | McKaughan and Macaraya (1967) |
| 133 | tbl | tbol1240 | Tboli (Tagabili) | Tboli_Tagabili_133 | Reid (1971) |
| 137 | tnt | tont1239 | Tontemboan | Tontemboan_137 | Karisoh (2005) |
| 138 | tsu | tsou1248 | Tsou T63 | Tsou_T63_138 | Tung (1964) |
| 144 | mbb | west2555 | Western Bukidnon Manobo | Western_Bukidnon_Manobo_144 | Reid (1971) |
| 153 | ceb | cebu1242 | Cebuano | Cebuano_153 | Go and Hermelito (2005) |
| 157 | ivv | ivat1242 | Itbayaten | Itbayaten_157 | Reid (1971) |
| 177 | pwn | paiw1248 | Paiwan (Kulalao F82) | Paiwan_Kulalao_F82_177 | Ferrell (1982) |
| 183 | sxn | sang1336 | Sangir | Sangir_183 | Steller and Aebersold (1959) |
| 193 | hil | hili1240 | Hiligaynon | Hiligaynon_193 | Salcedo (2005) |
| 202 | bnn | bunu1267 | Bunun F69, Southern | Bunun_F69_Southern_202 | Ferrell (1969) |
| 203 | xnb | kana1286 | Kanakanabu F69 | Kanakanabu_F69_203 | Ferrell (1969) |
| 240 | mog | mong1342 | Bolaang Mongondow | Bolaang_Mongondow_240 | Dunnebier (1951) |
| 241 | ssf | thao1240 | Thao B96 | Thao_B96_241 | R. Blust (1996) |
| 248 | ivv | ivat1242 | Iraralay | Iraralay_248 | Tsuchida, Yamada, and Moriguchi (1989) |
| 249 | ivv | ivat1242 | Itbayat | Itbayat_249 | Tsuchida, Yamada, and Moriguchi (1989) |
| 250 | ivv | ivat1242 | Imorod | Imorod_250 | Tsuchida, Yamada, and Moriguchi (1989) |
| 251 | ivv | ivat1242 | Ivasay | Ivasay_251 | Tsuchida, Yamada, and Moriguchi (1989) |
| 252 | ivv | ivat1242 | Isamorong | Isamorong_252 | Tsuchida, Yamada, and Moriguchi (1989) |
| 254 | tao | yami1254 | Yami | Yami_254 | Ferrell (1969) |
| 255 | tay | atay1247 | Atayal - Squliq F69 | Atayal_Squliq_F69_255 | Ferrell (1969) |
| 256 | tay | atay1247 | Atayal - C’uli’ F69 (Bandai) | Atayal_Culi_F69_Bandai_256 | Ferrell (1969) |
| 260 | ckv | kava1241 | Kavalan F69 | Kavalan_F69_260 | Ferrell (1969) |
| 265 | plw | bata1301 | Palawan Batak | Palawan_Batak_265 | Reid (1971) |
| 266 | uun | kulo1237 | Pazih F69 | Pazih_F69_266 | Ferrell (1969) |
| 271 | pyu | puyu1239 | Puyuma (Chihpen F69) | Puyuma_Chihpen_F69_271 | Ferrell (1969) |
| 272 | dru | ruka1240 | Rukai (Budai F69) | Rukai_Budai_F69_272 | Ferrell (1969) |
| 273 | sxr | saar1237 | Saaroa F69 | Saaroa_F69_273 | Ferrell (1969) |
| 274 | xsy | sais1237 | Saisiyat F69 | Saisiyat_F69_274 | Ferrell (1969) |
| 275 | trv | taro1264 | Seediq F69 (Sakura) | Seediq_F69_Sakura_275 | Ferrell (1969) |
| 277 | tgl | taga1270 | Tagalog | Tagalog_277 | Llamzon (2005) |
| 279 | ibg | iban1267 | Ibanag | Ibanag_279 | Family (2005) |
| 288 | ilo | ilok1237 | Ilokano | Ilokano_288 | Blust (2005) |
| 289 | ivv | babu1242 | Babuyan | Babuyan_289 | Tsuchida, Yamada, and Moriguchi (1989) |
| 291 | bpr | koro1310 | Koronadal Blaan | Koronadal_Blaan_291 | Savage (1986) |
| 293 | bps | sara1326 | Sarangani Blaan | Sarangani_Blaan_293 | Savage (1986) |
| 335 | tao | yami1254 | Yami | Yami_335 | Rau (2005) |
| 350 | ami | amis1246 | Amis (Central) | Amis_Central_350 | R. A. Blust and Liu (2005) |
| 369 | byq | basa1287 | Basay TsYM91 | Basay_TsYM91_369 | Tsuchida and Yamada (1991) |
| 389 | tgl | taga1270 | Tagalog | Tagalog_389 | Luna (2006) |
| 405 | pag | pang1290 | Pangasinan | Pangasinan_405 | Daroya (2006) |
| 408 | tgl | taga1270 | Tagalog (Anthony dela Paz) | Tagalog_Anthony_dela_Paz_408 | Paz (2006) |
| 409 | agt | cent2084 | Agta | Agta_409 | Reid (1971) |
| 410 | att | pamp1244 | Atta, Pamplona | Atta_Pamplona_410 | Reid (1971) |
| 411 | blw | bala1310 | Balangaw | Balangaw_411 | Reid (1971) |
| 412 | bya | bata1301 | Batak, Palawan | Batak_Palawan_412 | Reid (1971) |
| 413 | bpr | koro1310 | Bilaan, Koronadal | Bilaan_Koronadal_413 | Reid (1971) |
| 414 | bps | sara1326 | Bilaan, Sarangani | Bilaan_Sarangani_414 | Reid (1971) |
| 415 | bkd | binu1244 | Binukid | Binukid_415 | Reid (1971) |
| 416 | lbk | guin1256 | Bontok, Guina-ang | Bontok_Guina-ang_416 | Reid (1971) |
| 417 | dgc | casi1235 | Dumagat, Casiguran | Dumagat_Casiguran_417 | Reid (1971) |
| 418 | gad | gadd1244 | Gaddang | Gaddang_418 | Reid (1971) |
| 419 | ifa | amga1235 | Ifugao, Amganad | Ifugao_Amganad_419 | Reid (1971) |
| 420 | ifb | bata1298 | Ifugao, Batad | Ifugao_Batad_420 | Reid (1971) |
| 421 | ify | kele1259 | Ifugao, Bayninan | Ifugao_Bayninan_421 | Reid (1971) |
| 422 | ilk | ilon1239 | Ilongot, Kakiduge:n | Ilongot_Kakidugen_422 | Reid (1971) |
| 423 | ibl | ibal1244 | Inibaloi | Inibaloi_423 | Reid (1971) |
| 424 | isd | isna1241 | Isneg, Dibagat-Kabugao-Isneg | Isneg_Dibagat-Kabugao-Isneg_424 | Reid (1971) |
| 425 | ivv | ivat1242 | Itbayaten | Itbayaten_425 | Reid (1971) |
| 426 | itb | bino1237 | Itneg, Binongan | Itneg_Binongan_426 | Reid (1971) |
| 427 | ivv | ivat1242 | Ivatan, Basco Dialect | Ivatan_Basco_Dialect_427 | Reid (1971) |
| 428 | kqe | kala1388 | Kalagan | Kalagan_428 | Reid (1971) |
| 429 | knb | lubu1243 | Kalinga, Guinaang, Lubuagan Dialect | Kalinga_Guinaang_Lubuagan_Dialect_429 | Reid (1971) |
| 430 | kak | kaya1320 | Kallahan, Kayapa Proper | Kallahan_Kayapa_Proper_430 | Reid (1971) |
| 431 | ify | kele1259 | Kallahan, Keleyqiq | Kallahan_Keleyqiq_431 | Reid (1971) |
| 432 | xnn | nort2877 | Kankanay, Northern | Kankanay_Northern_432 | Reid (1971) |
| 433 | mmn | mama1275 | Mamanwa | Mamanwa_433 | Reid (1971) |
| 434 | atd | atam1240 | Manobo, Ata (up-river) | Manobo_Ata_up-river_434 | Reid (1971) |
| 435 | atd | atam1240 | Manobo, Ata (down-river) | Manobo_Ata_down-river_435 | Reid (1971) |
| 436 | mbd | diba1242 | Manobo, Dibabawon | Manobo_Dibabawon_436 | Reid (1971) |
| 437 | mbi | ilia1236 | Manobo, Ilianen (Kibudtungan Dialect) | Manobo_Ilianen_Kibudtungan_Dialect_437 | Reid (1971) |
| 438 | mta | cota1241 | Manobo, Kalamansig Cotabato (Paril Dialect) | Manobo_Kalamansig_Cotabato_Paril_Dialect_438 | Reid (1971) |
| 439 | mbs | sara1327 | Manobo, Sarangani, Kayaponga Dialect | Manobo_Sarangani_Kayaponga_Dialect_439 | Reid (1971) |
| 440 | mbt | mati1250 | Manobo, Tigwa, Iglogsad Dialect | Manobo_Tigwa_Iglogsad_Dialect_440 | Reid (1971) |
| 441 | mbb | west2555 | Manobo, Western Bukidnon | Manobo_Western_Bukidnon_441 | Reid (1971) |
| 442 | msk | mans1262 | Mansaka | Mansaka_442 | Reid (1971) |
| 444 | sbl | boto1242 | Sambal, Botolan | Sambal_Botolan_444 | Reid (1971) |
| 445 | snl | sang1337 | Sangil, Saragani Islands | Sangil_Saragani_Islands_445 | Reid (1971) |
| 446 | sxn | sang1336 | Sangir, Tabukang Dialect | Sangir_Tabukang_Dialect_446 | Reid (1971) |
| 447 | syb | cent2089 | Subanun, Sindangan | Subanun_Sindangan_447 | Reid (1971) |
| 448 | suc | west2557 | Subanon, Siocon | Subanon_Siocon_448 | Reid (1971) |
| 449 | tbl | tbol1240 | Tagabili | Tagabili_449 | Reid (1971) |
| 450 | tgt | cent2090 | Tagbanwa, Aborlan Dialect | Tagbanwa_Aborlan_Dialect_450 | Reid (1971) |
| 451 | tbk | cala1258 | Tagbanwa, Kalamian, Coron Island Dialect | Tagbanwa_Kalamian_Coron_Island_Dialect_451 | Reid (1971) |
| 452 | tsg | taus1251 | Tausug, Jolo Dialect | Tausug_Jolo_Dialect_452 | Reid (1971) |
| 458 | ill | iran1262 | Iranun | Iranun_458 | S. Conklin and Shaiddin (2006) |
| 466 | sgd | suri1273 | Surigaonon | Surigaonon_466 | Parker (2006) |
| 467 | hil | hili1240 | Ilonggo | Ilonggo_467 | Enriquez, Bautista, and Jr (1949) |
| 483 | btw | butu1244 | Butuanon | Butuanon_483 | Jesus (2007) |
| 494 | plv | sout2916 | S.W. Palawano | SW_Palawano_494 | B. Davis (2007) |
| 496 | txs | tons1240 | Tonsea | Tonsea_496 | Ticoalu (2007) |
| 513 | war | wara1300 | Waray-Waray | Waray-Waray_513 | Rajki (2008) |
| 618 | blf | buol1237 | Buol | Buol_618 | Zobel (2009) |
| 635 | bzg | babu1240 | Babuza Ts82 | Babuza_Ts82_635 | Tsuchida (1982) |
| 636 | ppu | papo1239 | Hoanya Ts82 | Hoanya_Ts82_636 | Tsuchida (1982) |
| 637 | ppu | papo1239 | Papora Ts82 | Papora_Ts82_637 | Tsuchida (1982) |
| 638 | bzg | babu1240 | Taokas Ts82 | Taokas_Ts82_638 | Tsuchida (1982) |
| 657 | krj | kina1250 | Kinaray-a | Kinaray-a_657 | Genzola (2009) |
| 660 | cps | capi1239 | Capisano | Capisano_660 | Ricarte (2010) |
| 671 | ksc | sout2908 | Kalinga, Southern | Kalinga_Southern_671 | Anonymous (1980) |
| 672 | kmk | limo1248 | Kalinga, Limos | Kalinga_Limos_672 | Anonymous (1981) |
| 673 | ebk | cent2292 | Bontok, Eastern | Bontok_Eastern_673 | Fukuda and Fukuda (1981) |
| 678 | itv | itaw1240 | Itawis | Itawis_678 | Tharp and Natividad (1976) |
| 679 | ibl | ibal1244 | Ibaloi | Ibaloi_679 | Anton (2010) |
| 681 | kne | kank1243 | Kankanaey | Kankanaey_681 | Allen (2010) |
| 685 | mdh | magu1243 | Maguindanaon | Maguindanaon_685 | Sullivan (1986) |
| 686 | agv | remo1247 | Sinauna | Sinauna_686 | Yap (2009) |
| 687 | rol | romb1245 | Romblon | Romblon_687 | Yap (2009) |
| 688 | cyo | cuyo1237 | Cuyonon | Cuyonon_688 | Yap (2009) |
| 707 | tiy | tiru1241 | Tiruray | Tiruray_707 | Schlegel (1971) |
| 708 | bno | bant1288 | Bantoanon (Banton) | Bantoanon_Banton_708 | Kilgour and Hendrickson (1992);Gordon and Kilgour (1986) |
| 710 | ubl | buhi1243 | Bikol (Buhinon) | Bikol_Buhinon_710 | Olson, Ballenas, and Borromeo (2009) |
| 711 | kml | lowe1412 | Kalinga (Minangali) | Kalinga_Minangali_711 | Olson, Machlan, and Amangao (2008) |
| 713 | yog | yoga1237 | Yogad | Yogad_713 | P. W. Davis and Mesa (2000) |
| 720 | ckv | kava1241 | Kavalan LTs | Kavalan_LTs_720 | P. J. Li and Tsuchida (2006) |
| 722 | bto | irig1242 | Bikol (Rinconada) | Bikol_Rinconada_722 | Lobel and Bucad (2001) |
| 726 | bnn | bunu1267 | Bunun (Takituduh L88) | Bunun_Takituduh_L88_726 | P. J.-K. Li (1988) |
| 730 | duo | dupa1235 | Dupaningan Agta | Dupaningan_Agta_730 | Robinson (2008) |
| 742 | tay | atay1247 | Atayal - C’uli’ L04 (Mayrinax) | Atayal_Culi_L04_Mayrinax_742 | P. J.-K. Li (2004) |
| 755 | msb | masb1238 | Masbatenyo | Masbatenyo_755 | Wolfenden (2001) |
| 759 | pyu | puyu1239 | Puyuma (Nanwang Cq) | Puyuma_Nanwang_Cq_759 | Cauquelin (1991) |
| 760 | uun | kulo1237 | Pazih LTs (Auran) | Pazih_LTs_Auran_760 | P. J. Li and Tsuchida (2001) |
| 783 | war | wara1300 | Samar-Leyte | Samar-Leyte_783 | Tizon (1972) |
| 788 | tgl | taga1270 | Marinduque Tagalog | Marinduque_Tagalog_788 | Soberano (1980) |
| 799 | ami | amis1246 | Amis (Fata’an) | Amis_Fataan_799 | P. J.-K. Li (2004) |
| 800 | ami | amis1246 | Amis (Farang) | Amis_Farang_800 | P. J.-K. Li (2004) |
| 801 | ais | nata1254 | Sakizaya | Sakizaya_801 | P. J.-K. Li (2004) |
| 802 | tay | atay1247 | Atayal - C’uli’ L04 (Skikun) | Atayal_Culi_L04_Skikun_802 | P. J.-K. Li (2004) |
| 803 | bnn | bunu1267 | Bunun (Takituduh L04) | Bunun_Takituduh_L04_803 | P. J.-K. Li (2004) |
| 804 | bnn | bunu1267 | Bunun (Takbanuaz) | Bunun_Takbanuaz_804 | P. J.-K. Li (2004) |
| 805 | bnn | bunu1267 | Bunun (Iskubun) | Bunun_Iskubun_805 | P. J.-K. Li (2004) |
| 806 | pwn | paiw1248 | Paiwan (Butanglu L04) | Paiwan_Butanglu_L04_806 | P. J.-K. Li (2004) |
| 807 | pwn | paiw1248 | Paiwan (Stimul L04) | Paiwan_Stimul_L04_807 | P. J.-K. Li (2004) |
| 808 | pwn | paiw1248 | Paiwan (Tjubar L04) | Paiwan_Tjubar_L04_808 | P. J.-K. Li (2004) |
| 809 | pyu | puyu1239 | Puyuma (Pilam L04) | Puyuma_Pilam_L04_809 | P. J.-K. Li (2004) |
| 810 | pyu | puyu1239 | Puyuma (Lower Pinlang L04) | Puyuma_Lower_Pinlang_L04_810 | P. J.-K. Li (2004) |
| 811 | pyu | puyu1239 | Puyuma (Katipul L04) | Puyuma_Katipul_L04_811 | P. J.-K. Li (2004) |
| 812 | dru | ruka1240 | Rukai (Tanan L04) | Rukai_Tanan_L04_812 | P. J.-K. Li (2004) |
| 813 | dru | ruka1240 | Rukai (Budai L04) | Rukai_Budai_L04_813 | P. J.-K. Li (2004) |
| 814 | dru | ruka1240 | Rukai (Maga L04) | Rukai_Maga_L04_814 | P. J.-K. Li (2004) |
| 815 | dru | ruka1240 | Rukai (Tona) | Rukai_Tona_815 | P. J.-K. Li (2004) |
| 816 | dru | ruka1240 | Rukai (Mantauran L04) | Rukai_Mantauran_L04_816 | P. J.-K. Li (2004) |
| 817 | xsy | sais1237 | Saisiyat L04 (Tungho) | Saisiyat_L04_Tungho_817 | P. J.-K. Li (2004) |
| 818 | xsy | sais1237 | Saisiyat L04 (Ta’ai) | Saisiyat_L04_Taai_818 | P. J.-K. Li (2004) |
| 819 | tay | atay1247 | Atayal - Squliq L04 | Atayal_Squliq_L04_819 | P. J.-K. Li (2004) |
| 820 | trv | taro1264 | Seediq L04 (Paran) | Seediq_L04_Paran_820 | P. J.-K. Li (2004) |
| 821 | trv | taro1264 | Seediq L04 (Toda) | Seediq_L04_Toda_821 | P. J.-K. Li (2004) |
| 822 | trv | taro1264 | Seediq L04 (Hecuo) | Seediq_L04_Hecuo_822 | P. J.-K. Li (2004) |
| 823 | trv | taro1264 | Seediq L04 (Truku) | Seediq_L04_Truku_823 | P. J.-K. Li (2004) |
| 824 | tsu | tsou1248 | Tsou (Duhtu L04) | Tsou_Duhtu_L04_824 | P. J.-K. Li (2004) |
| 825 | xnb | kana1286 | Kanakanabu L04 | Kanakanabu_L04_825 | P. J.-K. Li (2004) |
| 826 | sxr | saar1237 | Saaroa L04 | Saaroa_L04_826 | P. J.-K. Li (2004) |
| 832 | byq | basa1287 | Basay L04 | Basay_L04_832 | P. J. Li ([2004]) |
| 859 | bcl | cent2087 | Bikol (Bisakol) | Bikol_Bisakol_859 | Gimoro (2011) |
| 860 | cgc | kaga1256 | Kagayanen | Kagayanen_860 | Huggins (2011) |
| 978 | itv | itaw1240 | Itawis | Itawis_978 | Barrias (2013) |
| 1200 | pam | pamp1243 | Kapampangan | Kapampangan_1200 | Balatbat (2015) |
| 1208 | bcl | cent2087 | Naga Bikol | Naga_Bikol_1208 | Geopoet (2015) |
| 1211 | atz | arta1239 | Arta | Arta_1211 | Kimoto (2015) |
| 1257 | sgb | maga1263 | Ayta Mag-antsi | Ayta_Mag-antsi_1257 | Storck and Storck (2005) |
| 1260 | atk | atii1237 | Inati (Nagpana) | Inati_Nagpana_1260 | Pennoyer (1986/1987) |
| 1262 | abp | aben1249 | Ayta Abellen | Ayta_Abellen_1262 | Stone (2008) |
| 1263 | abc | amba1267 | Ayta Ambala | Ayta_Ambala_1263 | Stone (2008) |
| 1264 | sgb | maga1263 | Ayta Mag-anchi | Ayta_Mag-anchi_1264 | Stone (2008) |
| 1265 | smk | boli1256 | Bolinao | Bolinao_1265 | Stone (2008) |
| 1266 | sbl | boto1242 | Botolan Sambal | Botolan_Sambal_1266 | Stone (2008) |
| 1267 | blx | magi1241 | Ayta Mag-indi | Ayta_Mag-indi_1267 | Stone (2008) |
| 1268 |  | tina1248 | Sambal | Sambal_1268 | Stone (2008) |
| 1269 | iry | iray1237 | Iraya | Iraya_1269 | Barbian (1977) |
| 1270 | alj | alan1249 | Alangan | Alangan_1270 | Barbian (1977) |
| 1271 | tdy | tady1237 | Tadyawan | Tadyawan_1271 | Barbian (1977) |
| 1272 | bku | buhi1245 | Batangan | Batangan_1272 | Barbian (1977) |
| 1273 | bku | buhi1245 | Buhid | Buhid_1273 | Barbian (1977) |
| 1274 | hnn | hanu1241 | Hanunoo | Hanunoo_1274 | Barbian (1977) |
| 1275 | btn | rata1245 | Ratagnon | Ratagnon_1275 | Barbian (1977) |
| 1278 | itv | itaw1240 | Malaweg | Malaweg_1278 | Reid (1971) |
| 1328 | pwn | paiw1248 | Paiwan (Tjatjigel Egli) | Paiwan_Tjatjigel_Egli_1328 | Egli (2002) |
| 1463 | inn | isin1239 | Isinay (Dupax) | Isinay_Dupax_1463 | Perlawan (2015) |
| 1467 | tgl | taga1270 | Mindoro Tagalog | Mindoro_Tagalog_1467 | Marticio (2017) |
| 1547 | loc | bula1256 | Bulalakawnon | Bulalakawnon_1547 | R. David Zorc (2018) |
| 1548 | loc | disp1238 | Inonhan (Dispoholnon) | Inonhan_Dispoholnon_1548 | R. David Zorc (2018) |
| 1549 | kyk | kama1363 | Kamayo | Kamayo_1549 | R. David Zorc (2018) |
| 1550 | clu | semi1263 | Caluyanun (Semirara) | Caluyanun_Semirara_1550 | R. David Zorc (2018) |
| 1551 | bgi | gian1241 | Guiangan (Sirib) | Guiangan_Sirib_1551 | R. David Zorc (2018) |
| 1552 | bgi | gian1241 | Guiangan (Baguio) | Guiangan_Baguio_1552 | R. David Zorc (1972) |
| 1553 | atk | atii1237 | Inati (Hamtic) | Inati_Hamtic_1553 | Hsiu (2018) |
| 1554 | atk | atii1237 | Inati (Jordan, Guimaras) | Inati_Jordan_Guimaras_1554 | Hsiu (2018) |
| 1555 | tgn | natu1247 | Tandaganon (Naturalis) | Tandaganon_Naturalis_1555 | R. David Zorc (1972) |
| 1569 | agn | agut1237 | Agutyanen | Agutyanen_1569 | Caabay and Melvin (2018) |
| 1635 | dul | alab1246 | Inagta Alabat | Inagta_Alabat_1635 | Lobel et al. (2020) |
| 1637 | sgd | suri1273 | Surigaonon | Surigaonon_1637 | Laude (2019) |
| 1638 | suc | west2557 | Western Subanon | Western_Subanon_1638 | Combi (2019) |
| 1641 | abd | cama1250 | Manide (Labo) | Manide_Labo_1641 | Doroja (2017) |

Allen, Janet. 2010. personal communication.

Anonymous. 1980. *A Topical Vocabulary in English, Pilipino, Ilocano, and Southern Kalinga*. Summer Institute of Linguistics.

———. 1981. *A Classified Vocabulary: English, Pilipino and Limos Kalinga*. Summer Institute of Linguistics.

Anton, Sofia Olga. 2010. *A Handy Guidebook to the Ibaloi Language*. Baguio City, Philippines: Tebtebba Foundation.

Balatbat, Eduardo John A. 2015. personal communication.

Barbian, Karl-Josef. 1977. *The Mangyan Languages of Mindoro*. Cebu City: University of San Carlos.

Barrias, Norman. 2013. personal communication.

Blust. 2005. personal communication.

Blust, Robert. 1996. “Some Remarks on the Linguistic Position of Thao.” *Oceanic Linguistics* 35 (2): 272–94.

Blust, Robert A., and Dorinda Liu. 2005. personal communication.

Caabay, Marilyn A., and Melissa S. Melvin. 2018. *Agutaynen - English Dictionary*. Webonary.org: Linguistic Society of The Philippines. <https://agutaynen.webonary.org/>.

Cauquelin, Josiane. 1991. “The Puyuma Language.” *Bijdragen Tot de Taal-, Land- En Volkenkunde* 147 (1): 17–60.

Combi, Jernan. 2019. personal communication.

Conklin, Harold C. 1953. *Hanunóo-English Vocabulary*. Vol. 9. University of California Publications in Linguistics. Berkeley; Los Angeles: University of California Press.

Conklin, Sean, and Mabulmaddin Haji Shaiddin. 2006. personal communication.

Daniega, Amy V. 2005. personal communication.

Daroya, Emerich. 2006. personal communication.

Davis, Bill. 2007. personal communication.

Davis, Philip W., and Angel D. Mesa. 2000. *A Dictionary of Yogad*. Vol. 17. Languages of the World : Dictionaries. München: Lincom Europa.

Doroja, D. M. A. 2017. “Amplifying Small Voices: A Grammar Sketch of Manide.” Master’s thesis, Diliman, Quezon City.: University of the Philippines Diliman.

Dunnebier, W. 1951. *Bolaang Mongondowsch-Nederlandsch Woordenboek: Met Nederlandsch-Bolaang Mongondowsch Register*. ’s Gravenhage: M. Nijhoff.

Egli, Hans. 2002. *Paiwan Wörterbuch: Paiwan-Deutsch, Deutsch-Paiwan*. Wiesbaden: Wiesbaden: Harrassowitz.

Enriquez, P. Jacobo, Jose A. Bautista, and Francis J. Jamolangue Jr. 1949. *Pocket Dictionary: English - Tagalog - Visayan (Cebuano - Ilongo) Vocabulary*. Manila: Philippine Book Co.

Family, Telan. 2005. personal communication.

Ferrell, Raleigh. 1969. *Taiwan Aboriginal Groups: Problems in Cultural and Linguistic Classification*. Monograph 17: Institute of Ethnology. Taiwan: Academica Sinica.

———. 1982. *Paiwan Dictionary. Pacific Linguistics, Series c-73*. Canberra: ANU.

Forman, M. L. 1971. *A Kapampangan Dictionary*. Honolulu: University of Hawaii.

Fukuda, Aiko, and Takashi Fukuda. 1981. *A Topical Vocabulary in English, Pilipino, Ilocano and Eastern Bontoc*. Manila: Summer Institute of Linguistics.

Genzola, Arnel. 2009. personal communication.

Geopoet. 2015. personal communication.

Gimoro, Roberto. 2011. personal communication.

Go, Gemma Losbaños, and Hermelito. 2005. personal communication.

Gordon, Ruth M., and Heather J. Kilgour. 1986. “Sociolinguistic Survey of Bantoanon.” *Studies in Philippine Linguistics* 6 (2): 1–93.

Hsiu, Andrew. 2018. Inati word lists.

Huggins, Andrea. 2011. personal communication.

Jesus, Vicente de. 2007. personal communication.

Karisoh, NY. J. A. 2005. personal communication.

Kilgour, Heather J., and Gail R. Hendrickson. 1992. “Bantoanon Phonology.” *Studies in Philippine Linguistics* 9 (1): 111–36.

Kimoto, Yukinori. 2015. personal communication.

Laude, Catharine Elizabeth. 2019. personal communication.

Li, Paul Jen-kuei. [2004]. *Selected Papers on Formosan Languages Taiwan Nan Dao Yu Yan Lun Wen Xuan Ji*. Vol. C3. Language and Linguistics Monograph Series. [Taipeh]: Institute of Linguistics, Academia Sinica.

Li, Paul Jen-Kuei. 1988. “A Comparative Study of Bunun Dialects.” *Bulletin of the Institute of History and Philology* 59 (2): 479–508.

———. 2004. “Basic Vocabulary for Formosan Languages and Dialects.” In *Selected Papers on Formosan Languages*, edited by Paul Jen-Kuei Li, 1483–1532. Taipei, Taiwan: Institute of Linguistics, Academia Sinica.

Li, Paul Jen-kuei, and Shigeru Tsuchida. 2001. *Pazih Dictionary Bazai Yu Ci Dian (Pazih Dictionary)*. Vol. Language and linguistics monographs series. Language and Linguistics Monographs Series; A2. Taipei: Institute of Linguistics (Preparatory office).

———. 2006. *Kavalan Dictionary*. Vol. 19. Language and Linguistics Monograph Series / a. Taipei, Taiwan: Inst. of Linguistics, Academia Sinica.

Llamzon, Ted. 2005. personal communication.

Lobel, Jason William, Amy Jugueta Alpay, Rosie Susutin Barreno, and Emelinda Jug Barreno. 2020. “Notes from the Field: Inagta Alabat: A Moribund Philippine Language, with Supporting Audio.” *Language Documentation & Conservation* 14: 1–57.

Lobel, Jason William, and Grace Uvero Bucad. 2001. *Rinconada Bikol-Filipino-English Phrasebook: With Mini-Dictionary*. Naga City, Philippines: Lobel & Tria Partnership.

Luna, Jose Miguel Vicente. 2006. personal communication.

Machmoed, Hamzah. 2005. personal communication.

Marticio, Dario. 2017. personal communication.

McFarland, Curtis D. 1977. *Northern Philippine Linguistic Geography*. Tokyo: Institute for the Study of Languages; Cultures of Asia; Africa.

McKaughan, Howard, and Batua A. Macaraya. 1967. *A Maranao Dictionary*. Honolulu: Univ. of Hawaii Press.

Olson, Kenneth S., Emy T. Ballenas, and Nilo M. and Borromeo. 2009. “Buhi’non (Bikol) Digital Wordlist: Presentation Form.” *Language Documentation & Conservation* 3 (2): 213–25.

Olson, Kenneth S., Glenn Machlan, and Nelson Amangao. 2008. “Minangali (Kalinga) Digital Wordlist: Presentation Form.” *Language Documentation & Conservation* 2 (1): 141–56.

Parker, Richard. 2006. personal communication.

Paz, Anthony dela. 2006. personal communication.

Pennoyer, Douglas F. 1986/1987. “Inati: The Hidden Negrito Language of Panay, Philippines.” *Philippine Journal of Linguistics* 18/19 (1986/1987): 1–36.

Perlawan, Sarah Eve. 2015. *Grammatical Sketch of Isinay Dupax*. Philippines: University of the Philippines, Diliman.

Rajki, Andras. 2008. personal communication.

Rau, D. Victoria. 2005. personal communication.

Reid, Lawrence Andrew. 1971. *Philippine Minor Languages: Word Lists and Phonologies*. Edited by Lawrence Andrew Reid. Vol. 8. Oceanic Linguistics Special Publication. [Honolulu]: Univ. of Hawaii Press.

Ricarte, Jose. 2010. personal communication.

Robinson, Laura C. 2008. “Dupaningan Agta: Grammar, Vocabulary, and Texts.” PhD thesis, Honululu: University of Hawai’i at Manoa; Univ. of Hawai’i. <http://www2.hawaii.edu/~lrobinso/Robinson-Dupaningan.pdf>.

Salcedo, Jamaica. 2005. personal communication.

Savage, Dale T. 1986. “A Reconstruction of Proto-Southern Mindanaon.” *Studies in Philippine Linguistics* 6 (2): 181–223.

Schlegel, Stuart A. 1971. *Tiruray-English Lexicon*. 1st ed. Vol. 67. University of California Publications in Linguistics. Berkeley: Los Angeles: University of California Press Berkeley.

Soberano, Rosa. 1980. *The Dialects of Marinduque Tagalog*. Vol. 69. Pacific Linguistics: Series b. [Canberra]: Dept. of Linguistics, Research School of Pacific Studies, Australian National University.

Steller, K. G. F., and W. E. Aebersold. 1959. *Sangirees-Nederlands Woordenboek*. s-Gravenhage: ’s-Gravenhage: Martinus Nijhoff.

Stone, Roger. 2008. “The Sambalic Languages of Central Luzon.” *Studies in Philippine Languages and Cultures* 19: 158–83.

Storck, Margaret, and Kurt Storck. 2005. *Ayta Mag-Antsi-English Dictionary*. Manila: Summer Institute of Linguistics, Philippines.

Sullivan, Robert E. 1986. *A Maguindanaon Dictionary: Maguindanaon-English, English-Maguindanaon*. Cotabato City, Philippines: Notre Dame University, Institute of Cotabato Cultures.

Tharp, James A., and Mateo C. Natividad. 1976. *Itawis-English Wordlist with English-Itawis Finderlist*. New Haven, Connecticut: Human Relations Area Files, Inc.

Theddon, L. 2005. personal communication.

Ticoalu, Billy S. C. 2007. personal communication.

Tizon, Alfonsa E. 1972. *Tagalog-Samar-Leyte Cognate Words with Identical and Different Meanings*. Manila: Institute of National Language (Philippines).

Tsuchida, Shigeru. 1982. *A Comparative Vocabulary of Austronesian Languages of Sinicized Ethnic Groups in Taiwan, Part i: Western Taiwan*. Vol. 7. Memoirs of the Faculty of Letters. Tokyo: University of Tokyo.

Tsuchida, Shigeru, and Yukihiro Yamada. 1991. “Ogawa’s Siraya/Makatao/Taivoan Comparative Vocabulary.” In *Linguistic Materials of the Formosan Sinicized Populations i: Siraya and Basai*, edited by Shigeru Tsuchida, Yukihiro Yamada, and Tsunekazu Moriguchi, 1–194. Tokyo: Department of Linguistics.

Tsuchida, Shigeru, Yukihiro Yamada, and Tsunekazu Moriguchi. 1989. *List of Selected Words of Batanic Languages*. Tokyo: University of Tokyo.

Tung, T’ung-Ho. 1964. *A Descriptive Study of the Tsou Language, Formosa*. Vol. 48. Special Publications. Taipei: Institute of History; Philology, Academia Sinica.

Usup, Hunggu Tadjuddin. 2005. personal communication.

Wolfenden, Elmer. 2001. *A Masbatenyo-English Dictionary*. Vol. 38. Philippine Journal of Linguistics : Special Monograph Issue. Manila: Linguistic Society of the Philippines.

Yap, Fe Aldave. 2009. *Rehiyong Tagalog: Lexicon Ng Mga Wika at Diyalekto*. Manila: University of Santo Tomas.

Zobel, Erik. 2009. personal communication.

Zorc, Dave. 2005. personal communication.

Zorc, R David. 2018. personal communication.

Zorc, R. David. 1972. Unpublished field notes.
